# Pyrroloquinoline Quinone Administration Alleviates Allergic Airway Inflammation in Mice by Regulating the JAK-STAT Signaling Pathway

**DOI:** 10.1155/2022/1267841

**Published:** 2022-10-29

**Authors:** Zhihui Min, Jiebai Zhou, Ruolin Mao, Bo Cui, Yunfeng Cheng, Zhihong Chen

**Affiliations:** ^1^Institute of Clinical Science, Zhongshan Hospital, Fudan University, Shanghai, China; ^2^Respiratory Division of Zhongshan Hospital, Shanghai Institute of Respiratory Disease, Fudan University, Shanghai, China

## Abstract

The current asthma therapies are inadequate for many patients with severe asthma. Pyrroloquinoline quinone (PQQ) is a naturally-occurring redox cofactor and nutrient that can exert a multitude of physiological effects, including anti-inflammatory and antioxidative effects. We sought to explore the effects of PQQ on allergic airway inflammation and reveal the underlying mechanisms. *In vitro*, the effects of PQQ on the secretion of epithelial-derived cytokines by house dust mite- (HDM-) incubated 16-HBE cells and on the differentiation potential of CD4+ T cells were investigated. *In vivo*, PQQ was administered to mice with ovalbumin- (OVA-) induced asthma, and lung pathology and inflammatory cell infiltration were assessed. The changes in T cell subsets and signal transducers and activators of transcription (STATs) were evaluated by flow cytometry. Pretreatment with PQQ significantly decreased HDM-stimulated thymic stromal lymphopoietin (TSLP) production in a dose-dependent manner in 16-HBE cells and inhibited Th2 cell differentiation *in vitro*. Treatment with PQQ significantly reduced bronchoalveolar lavage fluid (BALF) inflammatory cell counts in the OVA-induced mouse model. PQQ administration also changed the secretion of IFN-*γ* and IL-4 as well as the percentages of Th1, Th2, Th17, and Treg cells in the peripheral blood and lung tissues, along with inhibition the phosphorylation of STAT1, STAT3, and STAT6 while promoting that of STAT4 in allergic airway inflammation model mice. PQQ can alleviate allergic airway inflammation in mice by improving the immune microenvironment and regulating the JAK-STAT signaling pathway. Our findings suggest that PQQ has great potential as a novel therapeutic agent for inflammatory diseases, including asthma.

## 1. Introduction

Pyrroloquinoline quinone (PQQ) is an aromatic tricyclic o-quinone. It is a redox cofactor for several prokaryotic dehydrogenases [1, 2]. PQQ is not biosynthesized in mammals. Picomolar to nanomolar levels of PQQ have been found inhuman and rat tissues [3], and especially large amounts have been found in human milk [4] because of the widespread distribution of PQQ in dietary sources [5]. In the past few years, PQQ has been brought into focus due to its physiological properties that could be good for human health. Mounting evidence has revealed that PQQ can influence multitudinous physiological and biochemical processes; for example, it exhibits growth-promoting activity and antidiabetic, antioxidative, and neuroprotective effects [6]. Recent research observed that PQQ showed no toxicity orgenotoxicity after oral administration [7]. Therefore, oral PQQ supplementation is a promising approach for improvement of health status.

Asthma may represent the clinical manifestation of a unique form of chronic airway inflammation and is often associated with allergies. Glucocorticoids have potent anti-inflammatory actions and are the most effective anti-inflammatory agents for the treatment of asthma. However, asthma is a syndrome with many distinct and overlapping phenotypes. Most patients with severe asthma who take oral glucocorticoids or high doses of inhaled glucocorticoids have some degree of glucocorticoid resistance [8]. Within this subpopulation, some patients continue to exhibit poor asthma control despite the addition of long-acting beta agonists (LABAs), long-acting muscarinic antagonists (LAMAs), and leukotriene receptor antagonists (LTRAs) to their inhaled corticosteroid (ICS) regimens. Recent studies have focused on novel pathogenetic mechanisms of severe asthma, as elucidation of such mechanisms may facilitate the discovery of new therapeutic targets and pathways [9–11]. Immunomodulators such as anti-immunoglobulin E (IgE), anti-IL5, and anti-IL4 Ra antibodies have shown potential benefits in improving asthma control and reducing acute exacerbation in patients with severe asthma; however, each molecular-targeted medicine is limited to a specific genotype [12].

Targeting oxidative stress with antioxidant agents has been considered a novel strategy for lung disease treatment [13]. Given the anti-inflammatory and antioxidative properties of PQQ, we sought to explore the effects of PQQ on ovalbumin (OVA)-challenged allergic airway inflammation model mice and to reveal the underlying mechanisms in the current study.

## 2. Methods

### 2.1. Reagents and Antibodies

PQQ, purity ≥ 99.5%, was kindly donated by Changmao Biochemical Engineering Co., Ltd., Changzhou, China. OVA (S7951), aluminum hydroxide (AlOH_3_, A5253), and dexamethasone (DXM, D1756) were all bought from Sigma-Aldrich (St. Louis, MO, USA). Human TSLP ELISA Kit (CHE0059), human IL-33 ELISA Kit (CHE0034), mouse IL-4 ELISA Kit (CME0005), and mouse IL-5 ELISA Kit (CME0023) were purchased from 4A Biotech Co., Ltd, Beijing, China. Mouse IFN-*γ* ELISA Kit (DY485-05) was purchased from R&D Systems, Inc. (Minneapolis, MN, USA). Collagenase IV was purchased from Worthington Biochemical Corporation (Lakewood, USA). A PQQ stock solution (10 mM) was dissolved in sterile PBS, filtered and stored at -20°C.

### 2.2. Animals

Six- to eight-week old female Balb/c mice (weight range 19-24 g) were obtained from Shanghai SLAC Laboratory Animal Company. The animals were given a sterile diet and water and kept in individually ventilated cages to avoid infection at 22°C room temperature and controlled 50% ± 5% humidity, with 12 h day and night switching. The study was met with approval by the Regulations of the Animal Ethics Committee of Zhongshan Hospital, Fudan University (No. 2018-035).

### 2.3. 16-HBE Cell Viability and Cytokine Production Assays

16-HBE cells were treated with different concentrations of PQQ for 24 h or 48 h. After that, cell viability was calculated with Cell Counting Kit-8 (CCK-8) according to the directions. PBS was added for the control group. For cytokine production experiments, 16-HBE cells were preincubated with different doses of PQQ or with 1 *μ*M DXM for 2 h. After stimulation with 1.13 *μ*g/ml house dust mites (HDMs) for 24 h, the supernatant was collected for ELISA.

### 2.4. Th2 Cell Differentiation

CD4 + T cells in mouse spleens purified by magnetic beads were cultured towards Th2-inducing conditions with recombinant murine IL-4 (3 ng/ml), rhIL-2 (50 U/ml) (mouse cross-reactive), anti-mouse CD3 antibody (1.5 mg/ml), and anti-mouse CD28 antibody (0.5 mg/ml). In control group, the CD4 + T cells were cultured at the same processing conditions but without IL-4. Different doses of PQQ and 1 *μ*M DXM were also added to the culture solution. The CD4 + T cells were primed for 5 days. At last, after phorbol 12-myristate 13-acetate (PMA) and ionomycin stimulation overnight, the supernatant of every group was collected for ELISA.

### 2.5. Generation of Mice with OVA-Induced Asthma and Administration of PQQ after Sensitization

A total of twenty-five female Balb/c mice were randomly separated into five research groups (*n* = 5 per group): the 0.9% normal saline control group, the OVA group, the OVA+DXM (1 mg/kg) group, the OVA+10 mg/kg PQQ group, and the OVA+20 mg/kg PQQ group. Except for those in the saline control group, the mice were sensitized with intraperitoneal injections of 100 *μ*l of saline suspension containing OVA (100 *μ*g) and AlOH_3_(2 mg) on days 0 and 7, respectively, and afterwards conducted with 50 *μ*l of PBS containing 100 *μ*g of OVA intranasally (i.n.) once a day from days 14 to 18. The mice in the control group carried out intraperitoneal injections of 100 *μ*l of 0.9% normal saline and nasal drops of 50 *μ*l of PBS. PQQ was dissolved in PBS at concentrations of 10 mg/kg and 20 mg/kg and administered by intraperitoneal injection two hours before every OVA challenge from days 8 to 18. The OVA+DXM group, as the positive control group, was treated in the same way as the PQQ group but with DXM instead of PQQ. Anesthesia was administered 24 h after the last medication. At last, mice were executed by cervical dislocation.

### 2.6. Bronchoalveolar Lavage Fluid (BALF) Collection and Inflammatory Cell Counting

The mice were anesthetized, and BALF was collected, and then immediately euthanized within 24 h after the last OVA treatment or OVA+PQQ/DXM administration. Firstly, tracheotomies and tracheal cannulation were performed with ketamine (100 mg/kg)-anesthetized mice by intraperitoneal injection, and three successive instillations of ice-cold PBS (total 0.5 ml) into the partial lobes were conducted. BALF was collected within half an hour and subsequently carefully collected the supernatant after centrifugation and froze at -80°C for detection of the IFN-*γ* and IL-4 cytokines. The total cell numbers in the remaining pellet were counted with an Automatic Blood Cell Analyzer (Mindray BC-5300Vet, Shenzhen Mindray Biomedical Electronics Co., Ltd.). Detailed differential cell counts in BALF were analyzed by flow cytometer (BD FACSAriaII) according to fluorescent staining of CD45, Ly6G, CD11c, and SiglecF. Serum and tissue samples were obtained for further analyses.

### 2.7. Assessment of Lung Histochemistry and Pathological Lung Injury

After collection of BALF, removed nonlavaged lobes and immediately put into 10% neutral formalin for 24 h. The lobes were dehydrated in increasing grades of ethanol, embedded in paraffin, serially sectioned (4 *μ*m) and stained with hematoxylin-eosin (H&E). They were observed and taken photographs under Olympus DP71 microscope. Histopathologic assessment of the lung sections was carried out, and the average peribronchial and perivascular inflammation scores were determined according to a scoring system described previously [14].

### 2.8. Elisa

Blood samples were collected, while the mice were sacrificed. Serums were collected by centrifugation and stored at -80°C. IgE levels were measured using a mouse IgE ELISA kit (ab157718, Abcam, Cambridge, MA, USA) on the basis of the company's recommendations. BALF obtained during the previous animal study were stored at -80°C.The cytokines of IL-4 and IL-5 were evaluated by ELISA kits (4A Biotech Co., Ltd, Beijing) according to the manufacturers' recommendations.

### 2.9. Measurement of Th1, Th2, Th17, and Treg Cell Populations in Blood and Lung Tissues by Flow Cytometry

Cells were isolated from blood samples and lung tissues. In short, the whole blood cells were lysed, washed, and resuspended in complete RPMI-1640 medium. White blood cells (WBCs) were distributed in 24-well plate (1 × 10^6^ cells/well) and incubated in stimulation solution containing 100 ng/ml PMA (Sigma-Aldrich, St. Louis, MO, USA), 1 *μ*g/ml ionomycin (Sigma-Aldrich, St. Louis, MO, USA), and 10 *μ*g/ml brefeldin A (BD Biosciences) for 6 h in the CO_2_ incubator. Detection of Th17 and Treg cells in our study did not require stimulation solution. After stimulation, the cells were first stained with the surface markers anti-CD4 FITC (Cat.No.553055, BD Pharmingen™) and anti-CD25 PE (Cat.No.558642, BD Pharmingen™) and were then fixed, permeabilized, and conducted intracellular staining with PE-conjugated anti-IFN-*γ* (Cat.No.554412, BD Pharmingen™), PE-conjugated anti-IL-4 (Cat.No.554435, BD Pharmingen™), ROR*γ*t (Cat.No.562682, BD Pharmingen™), and APC-conjugated FoxP3 antibodies (Cat.No.17-5773-80, eBioscience™) according to the manufacturers' instructions.

After BALF collection, the lung tissues were shredded with scissors and collagenase IV (1 mg/ml) digestion in RPMI-1640 medium with 5 unit/ml DNase I at 37°C with shaking for 1 h. The digested tissue mixtures were filtered by cell strainer and centrifuged. Discarded supernatants and pellets were lysed and washed. Later, the cell pellets were resuspended in complete medium, and the cells were distributed in 24-well plate with stimulants as described above. Finally, staining for flow cytometry was carried out as described above. Cell acquisition was performed on BD FACSAriaII cytometer, and the original data were analyzed using FlowJo7.6 software (TreeStar, Inc.)

### 2.10. Measurement of P-STAT1, P-STAT3, P-STAT4, and P-STAT6 Expression Levels in Lung Tissue and T Cells by Flow Cytometry

In order to elucidate the potential mechanisms of the reduced inflammation in the asthmatic model mice with PQQ treatment, we detected the effects of PQQ on central phosphorylated and total STAT proteins in lung tissue *in vivo* and in T cells *in vitro*. After BALF collection, the lobes of the lungs were shredded with scissors. The lung tissues were digested; the digests were filtered, and the cells were washed as described above. CD4 + T cells in normal mouse spleens purified by magnetic beads were seeded in 24-well plate and preincubated with 80 *μ*M PQQ or with 1 *μ*M DXM for 2 h. After stimulation with 50 *μ*M OVA for 24 h and control group, the cells were collected for the following staining. The cell pellets were resuspended in staining buffer (Cat. No. 554657, BD Pharmingen™) and then fixed and permeabilized with BD Cytofix™ Fixation Buffer (Cat. No. 554655, BD Biosciences) and Phosflow Perm Buffer III (Cat. No. 558050, BD Biosciences), respectively. Then, the isolated cells were intracellularly incubated with PE-conjugated mouse anti-stat1 (pY701) (Cat. No.612564, BD Phosflow™), PE-conjugated mouse anti-stat3 (pY705) (Cat.No.612569, BD Phosflow™), PE-conjugated mouse anti-stat4 (pY693) (Cat.No.558249, BDPhosflow™), PE-conjugated mouse anti-stat6 (pY641) (Cat.No.612701, BD Phosflow™), PE-conjugated mouse antitotal stat1 (Cat.No.558537, BD Phosflow™), PE-conjugated mouse anti-total stat3 (Cat.No.560391, BD Phosflow™), PE-conjugated mouse anti-total stat6 (Cat.No.560001, BD Phosflow™), mouse anti-stat4 monoclonal antibody(mab5287), and PE-conjugated rat anti-mouse IgG2A secondary antibody (Cat.No.F0129, R&D Systems), respectively, according to the manufacturers' recommendations. The results were detected on BD FACSAriaII cytometer, and the original data were analyzed using FlowJo7.6 software (TreeStar, Inc.).

### 2.11. Statistical Analysis

SPSS software version 13.0 was used to perform all analyses. Normality was assessed by the Shapiro-Wilk test. Difference between the control and treated group were analyzed by one-way analysis of variance (ANOVA) followed by a post hoc test. Data were denoted as the mean ± SEM. Statistical significance was determined by two-sided *p* values less than 0.05.

## 3. Results

### 3.1. Cell Viability and Cytokine Production in 16-HBE Cells Treated with Different Doses of PQQ

**Figure 1:**
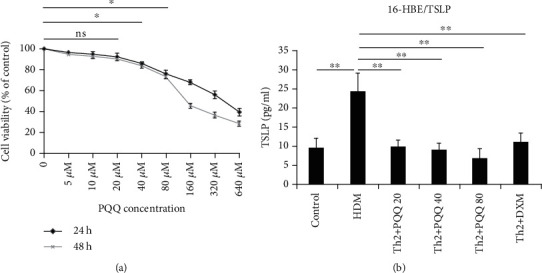
Cell viability and cytokine production in 16-HBE cells treated with different doses of PQQ. (a) 16-HBE cell viability was reduced after administration of PQQ in a dose-dependent manner. Cell viability was measured by CCK-8 assay after 24 h and 48 h of incubation with PQQ. (b) Cytokine production by 16-HBE cells after 2 h of pretreatment with PQQ and subsequent incubation with HDMs. The columns and error bars represent the means and SEMs (*n* = 3 per group). ^∗^*P* < 0.05, ^∗∗^*P* < 0.01; ns: not statistically significant. Similar results were obtained in three independent experiments.

To examine the cytotoxicity of PQQ, 16-HBE cells were incubated with various concentrations of PQQ for 24 h or 48 h. Cell viability was determined by CCK-8 assay. Normal untreated 16-HBE cells were used as the controls. The CCK-8 results demonstrated that treatment with PQQ reduced 16-HBE cell viability in a dose-dependent manner. 16-HBE cell viability was still more than 70% after 5 ~ 80 *μ*M PQQ treatment (Figure 1(a)). Based on these findings, the concentrations of PQQ used in the following ELISA ranged from 20 to 80 *μ*M.

Thymic stromal lymphopoietin (TSLP) is an epithelial-cell-derived cytokine. It plays an important role in initiating allergic inflammation [15]. To test the effects of PQQ on TSLP production in HDM-stimulated 16-HBE cells, cells pretreated with PQQ (20, 40, or 80 *μ*M) or DXM (1 *μ*M) were incubated with HDMs for 24 h. PQQ pretreatment for 2 h effectively dose-dependently reduced HDM-stimulated TSLP production in 16-HBE cells (Figure 1(b)). There was no effect of PQQ on IL-33 production in HDM-incubated 16-HBE cells (The figure was not shown).

### 3.2. PQQ Administration Inhibits Th2 Cell Differentiation

**Figure 2:**
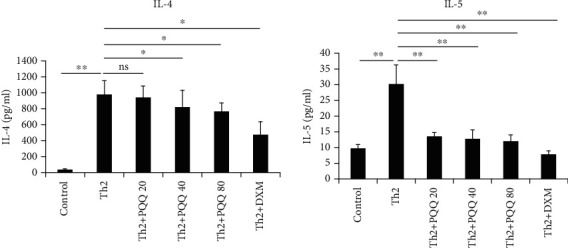
PQQ administration inhibits Th2 cell differentiation. CD4+ T cells were primed under Th2 conditions. PQQ and DXM were added. Five days later, the cells were stimulated with PMA and ionomycin. The Th2 cytokines were measured using an ELISA kit. The columns and error bars represent the means and SEMs (*n* = 3 per group). ^∗^*P* < 0.05, ^∗∗^*P* < 0.01; ns: not statistically significant. Similar results were obtained in three independent experiments.

To test the effect of PQQ on Th2 cell differentiation, we subjected CD4+ T cells to Th2 polarization *in vitro*. Th2 conditioned medium stimulated the production of the Th2 cytokines IL-4 and IL-5. As Figure 2 shows, PQQ administration significantly attenuated the production of IL-4 and IL-5 in a dose-dependent manner to an extent comparable to that of DXM treatment.

### 3.3. PQQ Administration Attenuates Airway Inflammation in an OVA-Induced Mouse Model

**Figure 3:**
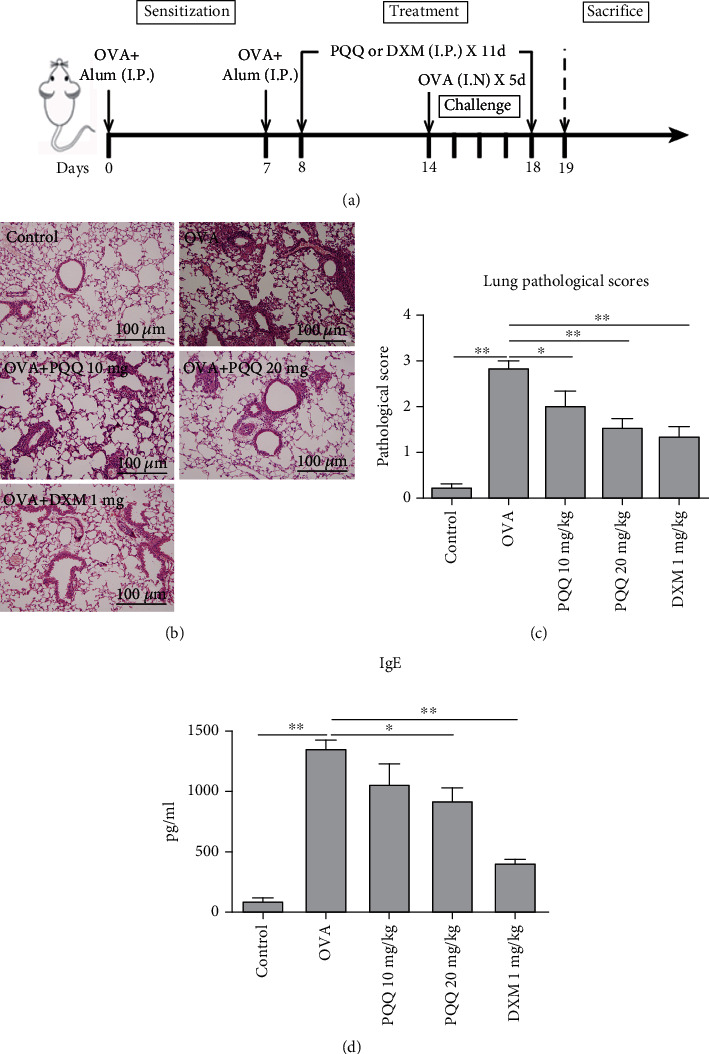
PQQ administration attenuates airway inflammation in an OVA-induced mouse model. (a) Protocols for establishment of the OVA-induced allergic asthma mouse model and administration of PQQ. (b) Representative photomicrographs of lung sections stained with H&E and examined at 100x magnification. (c) Lung pathological scores of peribronchial and perivascular inflammatory cell infiltration in asthmatic model mice and PQQ-/DXM-treated asthmatic model mice. (d) Serum IgE levels in asthmatic model mice and PQQ-/DXM-treated asthmatic model mice. The columns and error bars represent the means and SEMs (*n* = 5 per group). ^∗^*P* < 0.05, ^∗∗^*P* < 0.01. Similar results were obtained in at least three independent experiments.

To examine the effect of PQQ on airway inflammation *in vivo*, OVA was used to establish an allergic airway inflammation model with Balb/c mice. As described, mice were sensitized with OVA from days 0 to 7 and subsequently challenged with OVA from days 14 to 18. From days 8 to 18, mice were treated with PQQ at a dose of 10 mg/kg or 20 mg/kg (Figure 3(a)). The histopathological changes in lung tissue samples were apparently improved in PQQ-treated allergic airway inflammation model mice (Figure 3(b)). In addition, the lung pathological scores of peribronchial and perivascular inflammatory cell infiltration were significantly and dose-dependently decreased in the PQQ-treated groups (Figure 3(c)). As shown in Figure 3(d), PQQ significantly attenuated serum IgE upregulation at a dose of 20 mg/kg.

### 3.4. PQQ Administration Reduces BALF Inflammatory Cell Counts and Changes the Secretion of IFN-*γ* and IL-4

**Figure 4:**
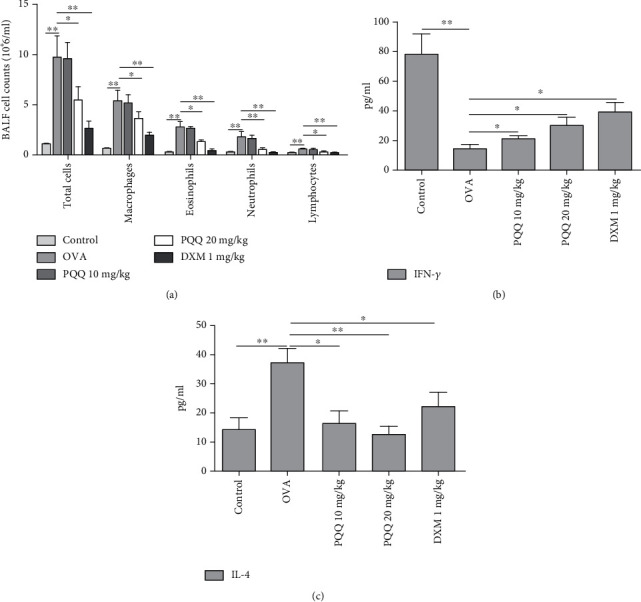
PQQ administration reduces BALF inflammatory cell counts and changes IFN-*γ* and IL-4 secretion. (a) Total cell numbers in BALF were counted with an Automatic Blood Cell Analyzer and specific differential cell counts in BALF were tested by flow cytometer. (b) Levels of released IFN-*γ* in BALF as measured by ELISA. (c) Levels of released IL-4 in BALF measured by ELISA. The columns and error bars represent the means and SEMs (*n* = 5 per group). ^∗^*P* < 0.05, ^∗∗^*P* < 0.01. Similar results were obtained in at least three independent experiments.

PQQ treatment at a dose of 20 mg/kg and DXM group significantly decreased total cell counts in the BALF of allergic airway inflammation model mice (Figure 4(a)). The affected cells included macrophages, eosinophils, neutrophils, and lymphocytes. PQQ administration also significantly and dose-dependently increased the secretion of IFN-*γ* (Figure 4(b)) and decreased the secretion of IL-4 (Figure 4(c)) in the BALF of allergic airway inflammation model mice. The level of IL-4 in the OVA+20 mg/kg PQQ group was even lower than that in the OVA+DXM group.

### 3.5. Effects of PQQ Treatment on Th1, Th2, Th17, and Treg Cells in Peripheral Blood and Lung Tissues of Asthmatic Model Mice

**Figure 5:**
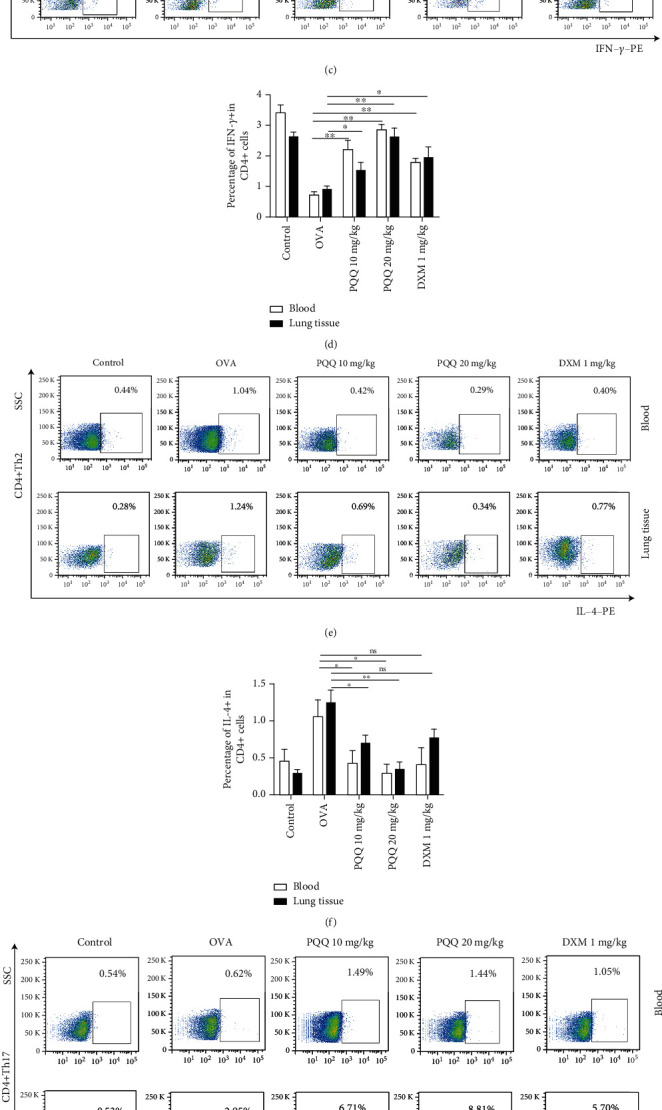
Effects of PQQ treatment on Th1, Th2, Th17, and Treg cells in peripheral blood and lung tissues of asthmatic model mice. (a and b) The gating strategy of flow cytometry for blood and tissue. (c and d) Representative scatter diagrams in flow cytometry and quantitative analysis of the percentages of CD4 + IFN-*γ* + T cells in blood and lung tissues. (e and f) Representative scatter diagrams in flow cytometry and quantitative analysis of the percentages of CD4 + IL-4+ T cells in blood and lung tissues. (g and h) Representative scatter diagrams in flow cytometry and quantitative analysis of the percentages of CD4 + ROR*γ*t+ T cells in blood and lung tissues. (i and j) Representative scatter diagrams in flow cytometry and quantitative analysis of the percentages of CD4 + CD25 + FoxP3+ T cells in blood and lung tissues. The columns and error bars represent the means and SEMs (*n* = 5 per group). ^∗^*P* < 0.05, ^∗∗^*P* < 0.01. Similar results were obtained in at least three independent experiments.

Flow cytometry assays were performed to examine the Th1, Th2, Th17, and Treg cell populations in both the peripheral blood and lung tissues of allergic airway inflammation model mice. As shown in Figure 5, PQQ treatment significantly increased the percentages of Th1 cells among total CD4+ T cells in the peripheral blood and lung tissues of allergic airway inflammation model mice in a dose-dependent manner. We noted that the percentages of Th1 cells in peripheral blood were higher than the same group in lung tissues and the DXM group (Figures 5(c) and 5(d)). In contrast, PQQ treatment significantly decreased the percentages of Th2 cells among total CD4+ T cells in both the peripheral blood and lung tissues of allergic airway inflammation model mice (Figures 5(e)–5(f)). Furthermore, PQQ significantly increased the populations of Th17 cells (Figures 5(g) and 5(h)) in lung tissues, which was higher than that of DXM group. Lastly, PQQ increased Treg cell populations in the peripheral blood and lung tissues of allergic airway inflammation model mice, and its effects were superior to those of DXM group (Figures 5(i) and 5(j)).

### 3.6. Effects of PQQ Treatment on the P-STAT1, P-STAT3, P-STAT4, and P-STAT6 Signaling Pathways

**Figure 6:**
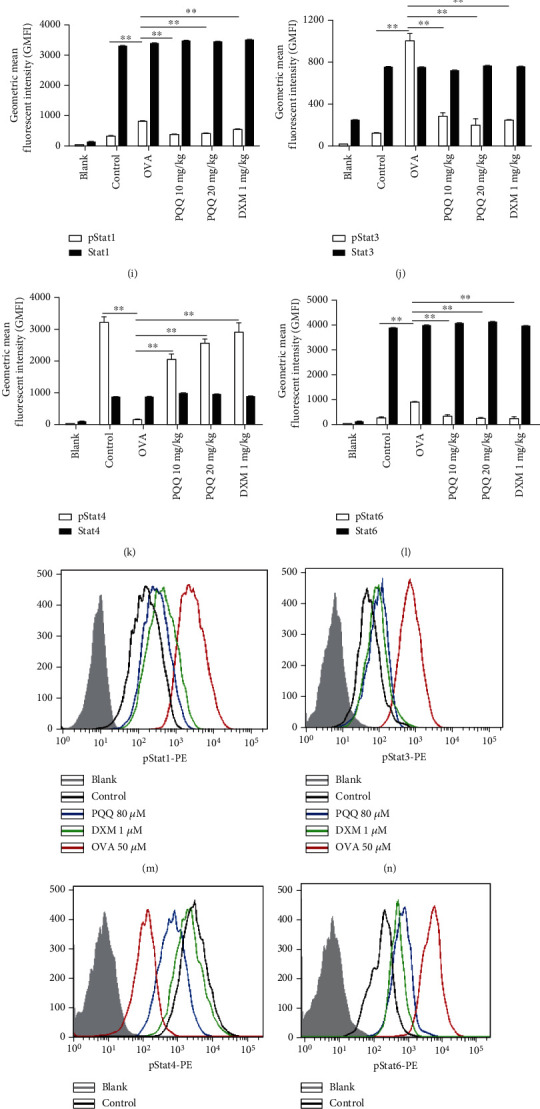
Effects of PQQ treatment on the pSTAT1, pSTAT3, pSTAT4, and pSTAT6 signaling pathways in lung tissue and T cells. The phosphorylated STAT1, STAT3, STAT4, and STAT6 levels in lung tissue (a–d), in T cells (M-P), and STAT1, STAT3, STAT4, and STAT6 levels in lung tissue (e–h) and in T cells (Q-T), which are presented as the geometric mean fluorescent intensity (GMFI) values measured by flow cytometry (colored lines). The gray peak is the geometric mean fluorescence intensity of the blank control. The quantitative analysis of the phosphorylated and total STAT proteins in mouse lung tissue (i–l) and in T cells (u–x). The columns and error bars represent the means and SEMs (*n* = 3 − 5 per group). ^∗∗^*P* < 0.01, ^∗^*P* < 0.05. Similar results were obtained in at least three independent experiments.

Activation of members of the STAT transcription family plays a significant role in inflammatory response associated with asthma. In this study, we employed flow cytometry assays to detect phosphorylation and total STAT subtypes. As predicted, compared with PBS exposure (in the control group), exposure to OVA significantly increased the phosphorylation of STAT1, STAT3, and STAT6 and decreased the phosphorylation of STAT4 in lung tissues (Figures 6(a)–6(d)). However, there were no difference in the total STAT1, STAT3, STAT4, and STAT6 levels among every group (Figures 6(e)–6(h)). In contrast, compared with OVA alone, PQQ treatment strongly inhibited the phosphorylation of STAT1 (Figures 6(a) and 6(i)), STAT3 (Figures 6(b) and 6(j)) and STAT6 (Figures 6(d) and 6(l)) in allergic airway inflammation model mice and promoted that of STAT4 (Figures 6(c) and 6(k)). PQQ exerted ameliorative effects similar to those of DXM. In addition, we detected the effect of PQQ on the same STAT subtypes in OVA-stimulated CD4 + T cells *in vitro* (Figures 6(m)–6(x)); the results were similar to those of lung tissue.

## 4. Discussion

Asthma is a chronic inflammatory airway disorder characterized by reversible airflow limitation and airway hyperresponsiveness. Intrinsic airway smooth muscle function abnormalities, airway remodeling in response to injury or inflammation, and interactions between epithelial and mesenchymal cells appear to modulate and add to the effects of airway inflammation to create the clinical manifestation of asthma. PQQ, a ubiquitous molecule, influences a multitude of biochemical and physiological processes through different mechanisms, including redox mechanisms, radical-scavenging mechanisms, and regulation of cell signaling pathways [16–19]. Previous data have shown that PQQ exerts potent immunosuppressive effects in the contexts of many diseases. For example, PQQ exhibits significant anti-neuro inflammatory activity in microglial cells by regulating the NF-ĸB and p38 MAPK signaling pathways [20]. In addition, PQQ decelerates rheumatoid arthritis progression by inhibiting inflammatory responses and joint destruction via modulation of the NF-ĸB and MAPK pathways [21]. We report, for the first time, that PQQ can also alleviate allergic airway inflammation in mice. Mice treated with PQQ demonstrated markedly attenuated peribronchial and perivascular inflammatory cell infiltration and improved lung pathological scores. Furthermore, therapeutic treatment with PQQ after OVA challenge obviously reduced IgE levels and total cell counts, including macrophages, eosinophils, neutrophils, and lymphocytes, in BALF. In addition, the critical epithelial derived cytokines, TSLP and IL-33 were regarded as indicators of Th2 airway inflammation [22]. To evaluate the effect of PQQ on the two cytokines *in vitro*, ELISA assays were tested. TSLP production in HDM-stimulated 16-HBE cells showed a decreasing tendency in dose-dependent manner after PQQ pretreatment for 2 h. But there was no inhibitory effect of PQQ on IL-33 production. It was reported [23] that under defective condition or signal, IL-33 could be quickly released from endothelial cells and played an early warning role to a certain extent. Maybe the time point in our study is a little bit long. We further confirmed the inhibition effect of PQQ on CD4+ T cells to Th2 polarization *in vitro,* which indicated decreasing production of IL-4 and IL-5 in a dose-dependent manner. These data showed that PQQ could reduce the key cytokines release in HDM-induced 16-HBE cell model and Th2 polarization *in vitro.*

Strong evidence indicates that T-cell activation is crucial to the initiation and maintenance of airway inflammation in patients with asthma [24]. Th1 cells play a critical role in the generation of delayed-type hypersensitivity responses, whereas Th2 cells can direct B cells to produce strong humoral reactions. Disruption of Th1/Th2 cell balance has traditionally been considered to be the key mechanism of asthma pathogenesis [25, 26]. IL-4, a Th2 cytokine, participates in the differentiation of uncommitted T cells into Th2 cells; the switching of B-lymphocyte immunoglobulin synthesis to IgE production; the selective endothelial cell expression of vascular cell adhesion molecule-1 (VCAM-1), which mediates eosinophil-, basophil-, and T cell-specific recruitment. IFN-*γ*, a defining cytokine of Th1 cells, promotes the activation of Th1 cells and inhibits naïve T cells from committing to a Th2 cell fate [27]. In this study, administration of PQQ significantly altered Th1 and Th2 cytokines, downregulating the release of IL-4 and upregulating the release of IFN-*γ*, with effects comparable to those of DXM. Furthermore, our data showed that PQQ-mediated immunoregulation of Th1 and Th2 cells was realized through upregulation of the Th1 cell population and downregulation of the Th2 cell population in the peripheral blood and lung tissues.

Th17 and Treg cells also play significant roles in the pathogenesis of asthma [28, 29]. Generally, the immunologic balance of Th17 and Treg cells helps to maintain immune homeostasis [30]. Upon allergen sensitization, Th17 cells enhance not only neutrophilic airway inflammation but also Th2 cell-mediated eosinophilic airway inflammation in mouse models of asthma [31, 32]. Treg cells help prevent immune activation, downregulating factors that trigger lesional inflammation [33]. Th17/Treg disequilibrium in asthma patients is closely related to the severity of asthma [34]. In our study, PQQ treatment promoted the expression of key transcription factor (ROR*γ*t) for Th17 cell and increased the Treg cell percentage in peripheral blood and lung tissues, exhibiting effects superior to those of DXM. These data further confirm that PQQ can regulate the Th1/Th2 and Th17/Treg equilibria.

The JAK-STAT signaling pathway has been shown to play a pivotal role in Th1 and Th2 differentiation [35]. STAT6 activation and STAT4 activation are significant events in the signaling cascades of IL-4 and IL-12, respectively. Given the importance of IL-4 and IL-12 in skewing Th cells toward either Th2 or Th1 subsets, STAT6 and STAT4 control multiple aspects of the Th differentiation program. Activation of STAT1 facilitates recruitment and activation of inflammatory cells mediated by upregulated expression of intercellular adhesion molecule 1 [36]. STAT3 is required for all features of asthma, including airway hyperresponsiveness and eosinophilia. In addition, STAT3 has been suggested to be critical for the production of certain chemokines in charge of the recruitment of inflammatory cells to the lungs during acute and chronic HDM allergen challenge [37]. Our data reveal that PQQ also regulates the JAK-STAT signaling pathway in allergic airway inflammation model mice by suppressing the phosphorylation activation of STAT1, STAT3, and STAT6 and triggering the phosphorylation activation of STAT4, exhibiting effects similar to those of DXM not only at CD4 + T cellular level but also in lung tissue.

Some patients with severe asthma do not achieve adequate control of their asthma with glucocorticoids despite adding LABAs, LAMAs, LTRAs, etc. to their ICS regimens. Patients with relative glucocorticoid resistance may respond better to nonglucocorticoid treatments than to glucocorticoid treatments. For this subpopulation, therapies with biological agents may be useful, such as anti-IgE therapy, anti-IL-5 therapy, and anti-IL-4/13 therapy. However, their high costs make these therapies inaccessible to some patients who need them. PQQ is a naturally occurring redox cofactor that serves as an essential nutrient and antioxidant. Our data suggest that PQQ administration alleviates allergic airway inflammation in mice by improving the immune microenvironment and regulating the JAK-STAT signaling pathway. Although the underlying mechanisms need to be further investigated, our findings provide insights that will aid in the development of more effective and less toxic agents for asthma control.

## 5. Conclusions

Our study demonstrates, for the first time, that PQQ can alleviate allergic airway inflammation in mice by improving the immune microenvironment and regulating the JAK-STAT signaling pathway. These findings suggest that PQQ has great potential as a novel therapeutic agent for inflammatory diseases, including asthma.

## Data Availability

The ovalbumin- (OVA-) challenged allergic airway inflammation model is currently widely used. All data generated during the study appear in the submitted article.
